# Orbital angular momentum transformation of optical vortex with aluminum metasurfaces

**DOI:** 10.1038/s41598-019-45727-6

**Published:** 2019-06-24

**Authors:** Yuchao Zhang, Xiaodong Yang, Jie Gao

**Affiliations:** Department of Mechanical and Aerospace Engineering, Missouri University of Science and Technology, Rolla, MO 65409 USA

**Keywords:** Metamaterials, Nanophotonics and plasmonics

## Abstract

The orbital angular momentum (OAM) transformation of optical vortex is realized upon using aluminum metasurfaces with phase distributions derived from the caustic theory. The generated OAM transformation beam has the well-defined Bessel-like patterns with multiple designed topological charges from −1 to +2.5 including both the integer-order and fractional-order optical vortices along the propagation. The detailed OAM transformation process is observed in terms of the variations of both beam intensity and phase profiles. The dynamic distributions of OAM mode density in the transformation are further analyzed to illustrate the conservation of the total OAM. The demonstration of transforming OAM states arbitrarily for optical vortex beams will lead to many new applications in optical manipulation, quantum optics, and optical communication.

## Introduction

Optical vortices possess helical phase fronts with azimuthal phase dependency of exp(*ilφ*), where *l* is the topological charge (TC) and *φ* represents the azimuthal angle, carrying orbital angular momentum (OAM) of *lħ* per photon. Based on the unique characteristics of optical vortices, numerous applications are implemented such as optical trapping^1–3^, optical communication^4,5^, quantum computation^6–10^, spin-orbit interaction^11,12^, and Bose-Einstein condensates^13,14^. The Laguerre-Gaussian (LG) beam propagating in free space has the invariant OAM value based on the OAM conservation law^15^. It is shown that TC inversion can be obtained by using the noncanonical optical vortex through the astigmatic lens^16,17^. In this case, the noncanonical optical vortex contains a superposition of different LG modes, while the OAM mode density is redistributed within the beam so that the overall OAM is still conserved. Besides, the OAM switching based on the superposition of multiple frozen waves has also been proposed^18,19^. However, the realization of arbitrary OAM transformation including both the high-order and fractional-order optical vortices has not been demonstrated yet. This is because the asymmetric deformations of noncanonical vortex structures are unstable for high-order TCs, so that the high-order noncanonical vortex will break apart into its corresponding single-charge constituents along the propagation direction^17^. Both the high-order and fractional-order optical vortices play important roles in the OAM-based applications. For example, in optical communication, multiple vortices of different TCs are used for OAM-multiplexed data encoding and decoding, while in quantum information processing, vortices with fractional TCs are used to test the entanglement of photons^20,21^. Therefore, it is essential to realize arbitrary OAM transformation containing both the high-order and fractional-order optical vortices.

Optical vortices can be generated by conventional spatial light modulators (SLMs)^22–24^, however, the phase modulation based on the optical path difference in the SLM varies as a function of wavelength due to the material dispersion, which limits the operation bandwidth. In recent years, plasmonic and dielectric metasurfaces have been widely used to tailor the phase, intensity and polarization of light^25–30^. Especially, metasurfaces with the phase modulation based on the geometric phase from polarization conversion provide an effective approach in making the integrated wavefront shaping devices with broadband operation, including vortex beam converters^31–35^, ultrathin optical lenses^36–41^, compact wave plates^42–45^, and multiplexed holograms^46–50^.

Based on the previously demonstrated TC inversion with plasmonic metasurfaces^51^, here, the OAM transformation of optical vortex beam is realized by using the ultrathin aluminum plasmonic metasurfaces containing nanoslit antennas with the phase distributions derived from the caustic theory. The proposed OAM transformation of vortex is realized by only a single metasurface with small area, which can be easily integrated into optical chips. Besides, the generated OAM transformation beam has nearly symmetric Bessel-like distributions for achieving OAM transformation with arbitrary integer and fractional TCs, including eight designed TCs from −1 to +2.5 with TC(*n*) = 0.5*n* − 1.5 (*n* = 1, 2, ∙∙∙, 8). The detailed TC transformation process is observed with the variations of both beam intensity and phase profiles. In particular, the horizontal dislocation cut lines for fractional-order vortices are obtained. The dynamic distributions of OAM mode density within the beam inner region (*r* < 5 µm) and the beam outer region (*r* > 5 µm) during the transformation process is further studied in Hilbert space constituted by LG modes, illustrating the OAM transformation rule and the conservation of the total OAM. Such demonstrated OAM transformation beam will have potential applications related to the high-order and fractional-order optical vortices. For example, OAM transformation beams can enable space-dependent optical tweezers for sorting and transporting particles, while the fractional-order optical vortices are useful for quantum information processing with OAM entanglement and OAM-multiplexed optical communication.

## Results

### Design of aluminum metasurface

**Figure 1:**
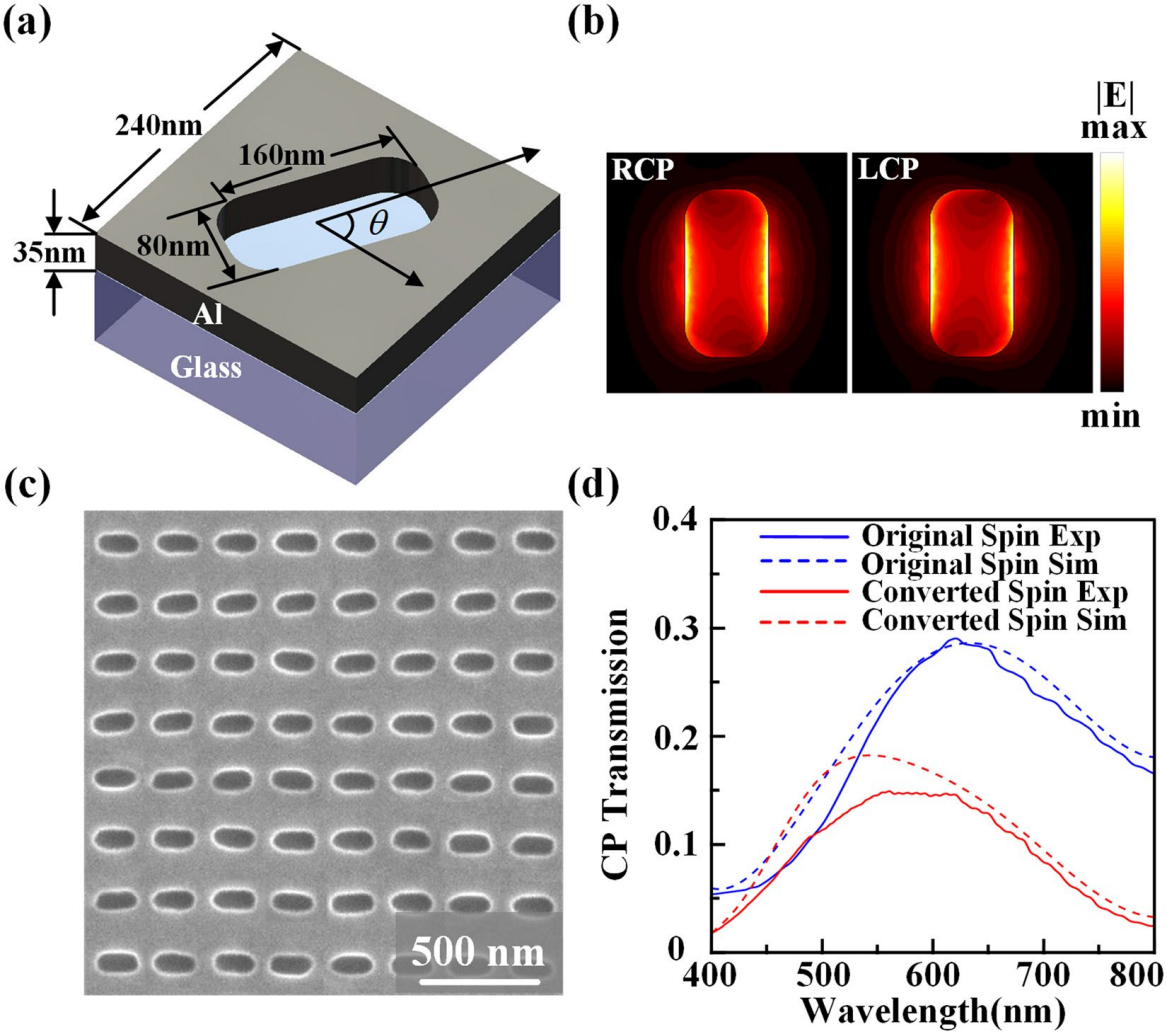
(**a**) The unit cell of nanoslit antenna at the rotated angle *θ*. (**b**) Calculated electric field |*E*| distributions of the nanoslit in circular polarizations at 532 nm. (**c**) A SEM picture of homogeneous nanoslit array. (**d**) Simulated and measured transmission spectra under circular polarization basis.

The designed metasurface with size of 120 µm × 120 µm constructed from nanoslit antenna arrays is fabricated on an aluminum film of 35 nm thick on glass substrate with focused ion beam (FIB) milling. Figure 1(a) shows the unit cell with a nanoslit antenna having a specific orientation angle *θ* to construct the metasurface. For each unit cell, the width is 80 nm, the length is 160 nm, and the period is 240 nm. As the circularly polarized light incidents on the nanoslit antenna, the converted spin component in transmission will acquire the geometric phase shift in 2*θ*. And the designed geometric phase distribution of the metasurface is realized by rotating the nanoslit at certain angles in each unit cell. The calculated electric field |E| distributions for the right- and left-handed circular polarizations (RCP and LCP) are shown in Fig. 1(b), showing pronounced polarization anisotropy. Figure 1(c) is the scanning electron microscope (SEM) picture of a homogeneous nanoslit array which is used to measure the transmission spectrum. Figure 1(d) shows the simulated and measured transmission spectra under circular polarizations. For LCP incident light onto the metasurface, the transmitted beam has the original spin component in LCP and the converted spin component in RCP. The original spin transmission is expressed as the intensity ratio of the transmitted original spin component and the incident original spin beam. The converted spin transmission is expressed as the intensity ratio of the converted spin component and the incident original spin beam. It is noticed that the converted spin component gets the maximum transmission of 15% close to the resonance wavelength of 550 nm.

The geometric phase profile used for generating the OAM transformation beam is derived from the caustic theory^52,53^. In this method, the caustic curves along convex trajectories are designed by modulating the phase on the initial input plane of *z* = 0. For the initial optical field at the input plane $\Psi ({x}_{0},{y}_{0},z=0)=G({r}_{0})\exp [ikQ({x}_{0},{y}_{0})]$, the geometric phase distribution specified into the metasurface is ${\phi }_{geom}=kQ({x}_{0},{y}_{0})$, where *k* = *2π/λ* represents the wavenumber. The optical field at the beam propagation distance of *z* is obtained by the paraxial Fresnel integral, as:

1 $$\Psi (x,y,z)=\frac{\exp (ikz)}{i\lambda z}{\rm{\iint }}G({r}_{0})\exp \{ik[Q({x}_{0},{y}_{0})+\frac{{(x-{x}_{0})}^{2}+{(y-{y}_{0})}^{2}}{2z}]\}d{x}_{0}d{y}_{0}$$

**Figure 2:**
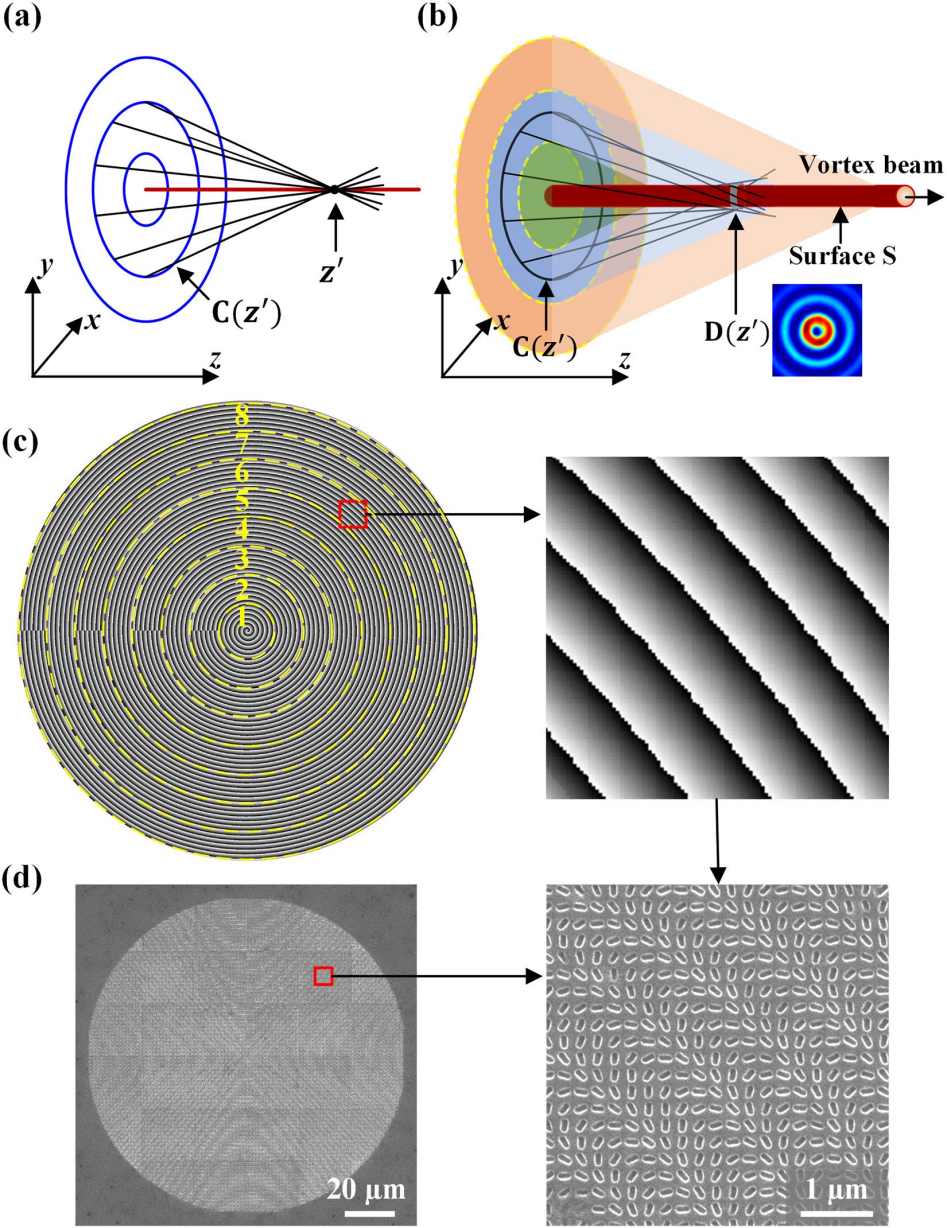
(**a**) The formation of caustic curve. (**b**) The caustic theory with the applied vortex structure into the initial optical field. The input plane is separated by yellow circles to several circular zones of different TCs. (**c**) The left figure is the phase distribution encoded on the metasurface with eight zones with different TC(*n*) = 0.5*n* − 1.5, and *n* = 1, 2, ∙∙∙, 8. The right figure is the magnified plot of the red rectangular region. (**d**) The left figure is the complete SEM picture of the entire fabricated metasurface, and the right figure is the magnified SEM picture of the red rectangular region.

According to the stationary phase approach, the optical field is calculated by the critical points satisfying both conditions of $(x-{x}_{0})/z=dQ/d{x}_{0}$ and $(y-{y}_{0})/z=dQ/d{y}_{0}$ on the input plane. As illustrated in Fig. 2(a), a point at the position of *z* = *z*′ corresponds a circle *C*(*z* = *z*′) located on the input plane, while the conical ray bundle radiated from such circle intersect at the point of *z* = *z*′. As the circle *C*(*z*) shifts and expands inside the input plane, the intersection locus of all conical ray bundles will form a continuous caustic curve. Here, the caustic curve is defined as $[f(z),g(z),z]$. The function *C*(*z*) for generating a conical ray bundle satisfies ${({x}_{0}-{x}_{c})}^{2}+{({y}_{0}-{y}_{c})}^{2}=R{(z)}^{2}$, where $({x}_{c},{y}_{c})$ is the coordinates of the circle center with ${x}_{c}=f-zf^{\prime}$ and ${y}_{c}=g-zg^{\prime}$, and *R*(*z*) is circle radius. This circle function *C*(*z*) is related to the propagation distance *z* with $z=z({x}_{0},{y}_{0})$. If no vortex is applied on the initial optical field, the conical ray bundle radiated from the circle *C*(*z*) is focused at the point $[f(z),g(z),z]$, as illustrated in Fig. 2(a). There are infinite number of rays passing through each point along the caustic curve, while only two rays will pass through the points outside the caustic curve. In other words, the total number of the incident rays changes abruptly from two to infinite along the *z* axis, this discontinuity property is the so-called catastrophe. The initial function of $Q({x}_{0},{y}_{0})$ is expressed by the formula^52^:

2 $$Q({x}_{0},{y}_{0})=\frac{{\rm{1}}}{2}{\int }_{0}^{z}\{{[f^{\prime} (\xi )]}^{2}+{[g^{\prime} (\xi )]}^{2}-{[\frac{R(\xi )}{\xi }]}^{2}\}d\xi -\frac{{(f-{x}_{0})}^{2}+{(g-{y}_{0})}^{2}}{2z}$$

By adding the vortex phase exp(*ilφ*), the initial input phase is exp[*i*(*kQ* + *lφ*)]. Then the corresponding stationary phase condition is $(l/k)\cdot d\phi /d{x}_{0}+dQ/d{x}_{0}=(x-{x}_{0})/z$ and $(l/k)\cdot d\phi /d{y}_{0}+dQ/d{y}_{0}=(y-{y}_{0})/z$. Due to the large wavenumber *k*, the terms (*l/k*)*∙dφ*/*dx*_0_ and (*l/k*)*∙dφ*/*dy*_0_ can be neglected, so that after adding the vortex structure, the optical field at *z* is still majorly contributed by the circle *C*(*z*). From the view of catastrophe theory, the rays radiated from *C*(*z*) are deflected by an angle of ∆**l = **(d*φ*/d*x*_0_, d*φ*/d*y*_0_)/*k* from the origin angle of **l = **(d*Q*/d*x*_0_, d*Q*/d*y*_0_), and at the plane *z* = *z*′ the intersection points of the bundle of light rays form a circle *D*(*z*′), as shown in Fig. 2(b). As *C*(*z*) varies on the input plane, the circle *D*(*z*) moves through the caustic curve to form a doubly-ruled surface **S**. No light ray exists inside the surface **S**, but two light rays intersect at each point outside the surface **S**, thus the number of light rays that enter into the surface **S** varies abruptly from zero to two to form a caustic surface. The produced vortex field with TC equals to that applied on *C*(*z*). By using polar coordinates of $({x}_{0},{y}_{0})={r}_{0}(\cos \,\phi ,\,\sin \,\phi )$ and $(x,y)=\rho (\cos \,\theta ,\,\sin \,\theta )$, the complex field at *z* is obtained by $F(z)=\int \exp (il\phi )\exp (-ik{r}_{0}\rho \,\cos (\phi -\theta )/z)d\phi \,=2\pi {J}_{l}(k{r}_{0}\rho /z)\exp (il\theta )$^52^, showing a vortex field with TC of *l* and the Bessel-like intensity distribution with order *l*. Since the achieved optical field has nearly symmetric Bessel-like intensity and phase profiles, this caustic method can be adopted to realize stable OAM transformation including arbitrary high-order and fractional-order optical vortices.

In order to design the OAM transformation beam, the input plane is divided into several circular zones, as shown in Fig. 2(b). Since the optical field distribution at *z* is mainly determined by *C*(*z*), vortex fields with different TCs at varying *z* can be created by several circular zones with the corresponding TCs. In Fig. 2(c), the input plane is divided into eight different circular zones featuring the TC changes in the order of {−1, −0.5, 0, +0.5, +1, +1.5, +2, +2.5}, which can be expressed as:

3 $${\rm{TC}}(n)=0.5n-1.5,\,{\rm{with}}\,\,n=1,\,2,\cdots ,8.$$

As the beam propagates, different circular zones will generate optical vortices with varying TCs with the sequence of TC(*n*) to realize the OAM transformation. The yellow dashed circles divide the input plane into eight separated circular zones, and the distance *Zt*(*n*) corresponding to each yellow dashed circle is defined as the transformation distance. The OAM transformation beam is designed to propagate along the straight line with the parameter function of [*f*(*z*) = 0, *g*(*z*) = 0, *z*]. *L*(z) is used to represent the TC at *z* as:

4 $$L(z)=\{{\rm{TC}}(n)|Zt(n-1) < z < Zt(n)\},\,{\rm{with}}\,n=1,\,2,\cdots ,8.$$

where TC(*n*) given by Eq. (3) and *Zt*(*n*) = 12*n* (µm). The input phase distribution is calculated firstly by using Eq. (2) to obtain $Q({x}_{0},{y}_{0})$, and then the vortex phase is added into $kQ({x}_{0},{y}_{0})$. The overall geometric phase distribution encoded on the metasurface can be expressed as:

5 $${\phi }_{geom}(x,y)=kQ({x}_{0},{y}_{0})+L[z({x}_{0},{y}_{0})]\arctan ({y}_{0}/{x}_{0}).$$

$Q({x}_{0},{y}_{0})$ is calculated with $f=g=f^{\prime} =g^{\prime} =0$ and the radius function *R*(*z*) = 0.625*z*. The circle function *C*(*z*) is ${x}_{0}^{2}+{y}_{0}^{2}=0.39{z}^{2}$, and thus $z({x}_{0},{y}_{0})=0.625\sqrt{{x}_{0}^{2}+{y}_{0}^{2}}.$ The function $Q({x}_{0},{y}_{0})=-0.625\sqrt{({x}_{0}^{2}+{y}_{0}^{2})}$ is obtained with Eq. (2). The wavenumber *k* = 11.8 at the wavelength $\lambda =0.532$µm, so that the total geometric phase is derived as:

6 $${\phi }_{geom}=-7.38\sqrt{({x}_{0}^{2}+{y}_{0}^{2})}+L(z)\arctan ({y}_{0}/{x}_{0}),$$

with $z({x}_{0},{y}_{0})=0.625\sqrt{{x}_{0}^{2}+{y}_{0}^{2}},$ as plotted in Fig. 2(c). The metasurface is constructed with each nanoslit rotated at the angle of $\theta ({x}_{0},{y}_{0})={\phi }_{geom}({x}_{0},{y}_{0})/2$. The SEM picture of the fabricated metasurface is displayed in Fig. 2(d), corresponding to Fig. 2(c).

The generation principle for the OAM transformation beam is different from the previously demonstrated OAM switching based on the frozen waves^18,19^. The frozen wave is generated by directly encoding the optical beam field into the SLM hologram, and a 4-*f* system is used to image the beam field onto the camera. In contrast, here, the OAM transformation beam is generated by engineering the phase profile on the Fourier transform plane, and the beam field is obtained by the Fourier transform of the initial phase profile, which is realized by Fresnel integral as the beam propagating in free space.

### OAM transformation of optical vortex

**Figure 3:**
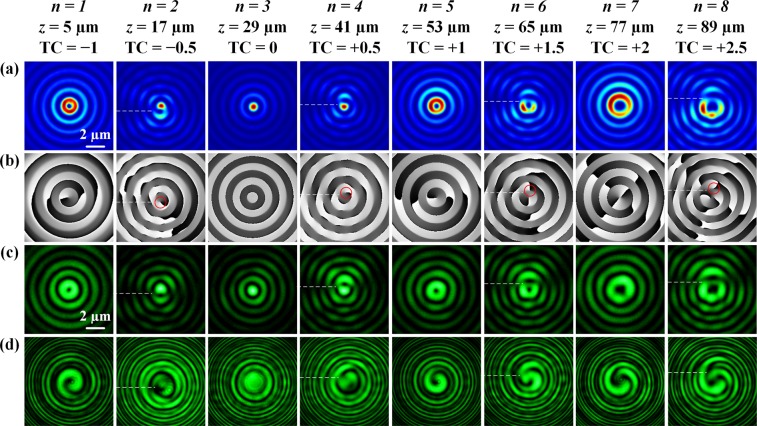
(**a**,**b**) Simulated beam intensity and phase profiles for the OAM transformation at *z*(*n*) = 12*n* − 7 (µm) with *n* = 1, 2, ∙∙∙, 8. (**c**) Measured beam intensity profiles and (**d**) interferometry patterns at 532 nm. The white dashed lines mark the dislocation cut lines of fractional-order vortices. The red circles in (**b**) mark the vortex structures located below or above the beam center of fractional-order vortices.

First, the OAM transformation of optical vortex is performed with numerical simulation by considering the Fresnel-Kirchhoff diffraction formula. Figure 3(a,b) plot the calculated intensity and phase distributions of the generated optical vortices with TCs of TC(*n*) = 0.5*n* − 1.5 at the propagation distances of

7 $$z(n)=12n-7(\mu m),{\rm{with}}\,n=1,2,\cdots ,8.$$

The TC is an integer of {−1, 0, +1, +2} for odd *n*, while the TC is a fractional number of {−0.5, +0.5, +1.5, +2.5} for even *n*. It shows from the intensity distributions that there are significant differences between the integer-order vortices and the fractional-order vortices, where the integer-order vortices display well-defined Bessel-like ring patterns, while the fractional-order vortices have a horizontal dislocation cut line (marked as the white dashed line) located along the negative *x*-axis. The phase profile at *z* = 5 µm (*n* = 1) exhibits one TC = −1 vortex structure at the beam center, having counterclockwise-increased phase. At *z* = 29 µm (*n* = 3), the vortex structure disappears giving zero TC. The phase profiles at *z* = 53 µm (*n* = 5) and *z* = 77 µm (*n* = 7) show vortex structures with clockwise-increased helical phases of 2π and 4π, corresponding to the TC of +1 and +2, respectively. The phase profiles for all the fractional-order vortices possess a horizontal phase dislocation cutline, where the vortex chain of alternating TC = +1 and TC = −1 is observed. According to the phase patterns at *z* = 17 µm (*n* = 2) and *z* = 41 µm (*n* = 4), besides the vortex chain, there is no vortex presented at the beam center but there is a vortex with TC of −1 or +1 located below or above the center, giving the vortex TC of −0.5 or + 0.5. At *z* = 65 µm (*n* = 6), one TC = +1 vortex is presented in the beam center together with another TC = +1 vortex located above the beam center, resulting in the TC = +1.5 vortex. Similarly, at *z* = 89 µm (*n* = 8) there are one TC = +2 vortex in the beam center and another TC = +1 vortex above the beam center, giving the TC = +2.5 vortex.

Next, the evolution of optical vortices during the OAM transformation process is measured at the wavelength of 532 nm. Figure 3(c) displays the measured beam intensity profiles at eight different *z*(*n*), and the Fig. 3(d) shows the interferometry patterns with a spherical wave. The evolution of intensity patterns along the beam propagation is coincident with the simulation results. For the integer-order vortices, the intensity distributions show well-defined Bessel-like ring-shape patterns with the predesigned TCs, while for the fractional-order vortices, there is a horizontal dislocation cut line located along the negative *x*-axis. The interference fringes of the integer-order vortices exhibit well-defined spirals. At *z* = 5 µm (*n* = 1), only one interference spiral with counterclockwise rotation presents, indicating the TC equals to −1. No interference spiral is obtained at *z* = 29 µm (*n* = 3), giving the TC of 0. And there are one or two interference spirals with clockwise rotation appear at *z* = 53 µm (*n* = 5) or *z* = 77 µm (*n* = 7), providing the TC equals to +1 or +2, respectively. All the interference patterns of the fractional-order vortices contain a vortex chain having the alternating TC = +1 and TC = −1 single vortices positioned through the cutline of phase discontinuity, which is marked by the white dashed line. Besides the peripheral vortex chain, the locations and charges of the measured interference spirals also agree with the simulated phase profiles for all the fractional-order vortices at *z* = 17 µm (*n* = 2), *z* = 41 µm (*n* = 4), *z* = 65 µm (*n* = 6), and *z* = 89 µm (*n* = 8).

**Figure 4:**
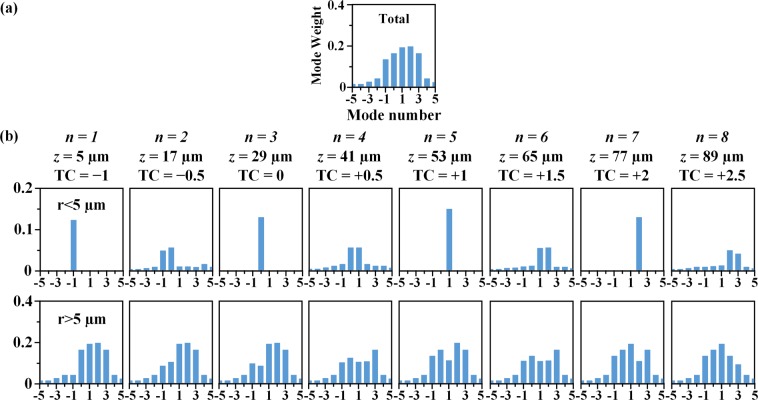
Calculated distributions of OAM mode at different *z*(*n*). (**a**) The total OAM mode distribution. (**b**) The dynamic distributions of OAM mode within the beam inner region of *r* < 5 µm (top row) and the beam outer region of *r* > 5 µm (bottom row).

The OAM mode analysis is further performed to understand the mechanism for OAM transformation of optical vortex. The total OAM is conversed during the evolution of OAM transformation process. It is known that the LG modes form a complete Hilbert set and have well-defined angular momentum, so the beam is decomposed into a superposition of LG modes with various OAM values, as illustrated in Fig. 4. The mode weight can be obtained by using the inner product of vortex field and LG modes: $${c}_{m}=\langle m|F(r,\phi )\rangle $$$$=\iint F(r,\phi )\exp (-im\phi )rdrd\phi $$, with the field distribution $$F(r,\phi )$$ and the eigenfunction $$|m\rangle =\exp (im\phi )$$^54^. The OAM mode is redistributed within the beam for the OAM transformation in free space^16^. The transverse beam cross section is divided into the inner region (*r* < 5 µm) and the outer region (*r* > 5 µm), and the OAM mode density redistributions between the two regions are plotted. Figure 4(a) shows the total OAM mode distribution, indicating that the OAM transformation is majorly contributed by the OAM states of $$|l=-1\rangle $$, $$|l=0\rangle $$, $$|l=+1\rangle $$, $$|l=+2\rangle $$ and $$|l=+3\rangle $$. Figure 4(b) shows the OAM mode distributions in the beam inner region and the outer region at different *z*(*n*). For the OAM mode distributions in the inner region, it is observed that each integer-order vortex contains almost only one pure OAM state of $$|l={\rm{TC}}(n)\rangle $$ with *n* = 1, 3, 5 or 7 and TC(*n*) = −1, 0, +1 or +2, while every fractional-order vortex is mainly composed of two OAM states $$|l={\rm{TC}}(n)-0.5\rangle $$ and $$|l={\rm{TC}}(n)+0.5\rangle $$ with *n* = 2, 4, 6 or 8 and TC(*n*) = −0.5, +0.5, +1.5, +2.5. The OAM transformation rule from the position of TC = *m* to the position of TC = *m* + 1 (*m* is an integer) can be described as the following. At the TC = *m* position, the OAM mode is mainly constituted by $$|l=m\rangle $$ state. As the vortex field is transformed to the TC = *m* + 0.5 position, the mode weight of $$|l=m\rangle $$ state decreases but that of $$|l=m+1\rangle $$ state increases to reach the nearly same weight. When the vortex is further transformed to the TC = *m* + 1 position, $$|l=m\rangle $$ mode disappears and the OAM mode is entirely constituted by $$|l=m+1\rangle $$ state. The OAM states in the outer region also vary accordingly to maintain the total OAM conversed. Such dynamic distribution of OAM mode density for the OAM transformation can benefit many OAM-based applications such as optical trapping, quantum information processing and optical communication.

**Figure 5:**
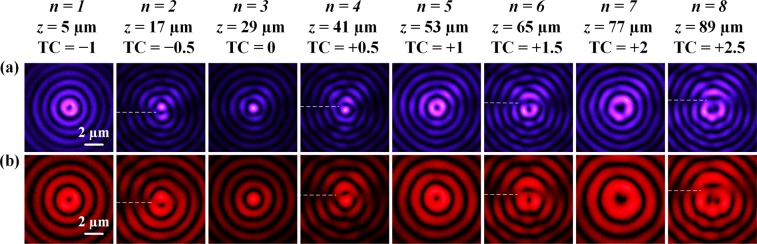
Measured beam intensity distributions for the OAM transformation process at different *z*(*n*) at (**a**) 405 nm and (**b**) 633 nm. The white dashed lines mark the dislocation cut lines of fractional-order vortices.

The broadband property of metasurface at the wavelengths of 405 nm and 633 nm is also studied. Figure 5 plots the measured beam intensity profiles of OAM transformation process at different *z*(*n*). At both wavelengths, the integer-order vortices possess Bessel-like ring-shape patterns, and the fractional-order vortices have horizontal dislocation cut lines. It is observed that the intensity distribution evolution is coincidence with the measured one at the wavelength of 532 nm, indicating the fabricated aluminum metasurface based on geometric phase can operate well across the entire visible wavelength range.

## Discussion

The OAM transformation with the capabilities of transforming arbitrary high-order and fractional-order optical vortices has been demonstrated with the aluminum metasurfaces designed from the theory of caustic surface and catastrophe. It is observed that the integer-order vortices have well-defined Bessel-like field distributions, while the fractional-order vortices have horizontal dislocation cut lines. The detailed OAM mode density redistribution in the beam inner region and outer region is analyzed in Hilbert space constituted by LG modes, in order to reveal the OAM transformation rule from $|m\rangle$ state to $|m+1\rangle$ state. The demonstrated OAM transformation will advance many potential applications related to optical vortices, for example, optical manipulation with spatially varying optical torques, quantum information processing with fractional OAM entanglement, and OAM-multiplexed communication.

## Methods

### Simulations

The CST Studio Suite package is employed to simulate the optical field distribution and the transmission spectrum. In the unit cell, periodic boundary conditions are used in *x* direction and *y* direction. The permittivity of aluminum is obtained from the spectroscopic ellipsometry, while the refractive index is 1.45 for glass substrate. The OAM transformation process plotted in Fig. 3(a,b) is calculated from the Fresnel-Kirchhoff integral:

8 $$\Psi (x,y,z)=\frac{1}{i\lambda }{{\rm{\iint }}}_{S}\Psi ({x}_{0},{y}_{0})[\frac{\cos (\overrightarrow{{\bf{n}}}{\boldsymbol{,}}{\bf{r}})-\,\cos (\overrightarrow{{\bf{n}}}{\boldsymbol{,}}{\bf{r}}{\boldsymbol{^{\prime} }})}{2}]\frac{{e}^{ikr}}{r}dS$$

where $\Psi ({x}_{0},{y}_{0})$ is the complex amplitude at the plane of *z* = 0 with *S* as the surface area and $\overrightarrow{n}$ as the surface normal, **r′** is the vector from the source point to a point at the plane of *z* = 0, **r** is the vector from the point at the plane of *z* = 0 to a point at the plane of *z*, *k* = 2π/*λ* represents the wavenumber.

### Sample fabrication

An aluminum film of 35 nm thick is deposited on glass substrate with electron-beam evaporation. The nanoslit arrays are then etched in the aluminum layer with focused ion beam (FEI Helios Nanolab 600, 30 kV, 9.7 pA). The metasurface contains 500 × 500 unit cells with the nanoslit size of 160 nm × 8  nm at the specified rotation angle.

### Optical characterization

The metasurface transmission spectra with incident circular polarizations in Fig. 1(d) are measured from a white light source, with the combined linear polarizer and quarter-wave plate to get the circularly polarized beam. The beam is focused on the metasurface by a 50× objective lens and the transmission spectrum is recorded by a spectrometer (Horiba, iHR 550) through a 10× objective lens. A glass substrate is used for normalizing the transmission spectrum. The metasurface operates across the broadband range of 400 nm to 800 nm, so three diode lasers at 405 nm, 532 nm and 633 nm are utilized. The intensity profiles and interferometry patterns of optical vortices are imaged by one 20× objective lens, one 0.5× tube lens and a color charge-coupled device (CCD) camera mounted on a linear translation stage.
